# Spectroscopic evidence of symmetry breaking in the superconducting vortices of UTe_2_

**DOI:** 10.1093/nsr/nwaf267

**Published:** 2025-07-04

**Authors:** Zhongzheng Yang, Fanbang Zheng, Dingsong Wu, Bin-Bin Zhang, Ning Li, Wenhui Li, Chaofan Zhang, Guang-Ming Zhang, Xi Chen, Yulin Chen, Shichao Yan

**Affiliations:** State Key Laboratory of Quantum Functional Materials, School of Physical Science and Technology, ShanghaiTech University, Shanghai 201210, China; State Key Laboratory of Quantum Functional Materials, School of Physical Science and Technology, ShanghaiTech University, Shanghai 201210, China; Department of Physics, University of Oxford, Oxford OX1 3PU, UK; Nanhu Laser Laboratory, Changsha 410073, China; Nanhu Laser Laboratory, Changsha 410073, China; State Key Laboratory of Quantum Functional Materials, School of Physical Science and Technology, ShanghaiTech University, Shanghai 201210, China; ShanghaiTech Laboratory for Topological Physics, ShanghaiTech University, Shanghai 201210, China; Nanhu Laser Laboratory, Changsha 410073, China; State Key Laboratory of Quantum Functional Materials, School of Physical Science and Technology, ShanghaiTech University, Shanghai 201210, China; State Key Laboratory of Low-Dimensional Quantum Physics, Department of Physics, Tsinghua University, Beijing 100084, China; State Key Laboratory of Quantum Functional Materials, School of Physical Science and Technology, ShanghaiTech University, Shanghai 201210, China; Department of Physics, University of Oxford, Oxford OX1 3PU, UK; ShanghaiTech Laboratory for Topological Physics, ShanghaiTech University, Shanghai 201210, China; State Key Laboratory of Quantum Functional Materials, School of Physical Science and Technology, ShanghaiTech University, Shanghai 201210, China; ShanghaiTech Laboratory for Topological Physics, ShanghaiTech University, Shanghai 201210, China

**Keywords:** superconducting vortex, symmetry breaking, spin-triplet superconductor, UTe_2_

## Abstract

The recently discovered heavy-fermion superconductor, UTe_2_, is an excellent candidate for spin-triplet superconductors in which electrons form spin-triplet Cooper pairs with spin *S* = 1 and odd parity. Unconventional superconductivity often hosts unconventional vortices. Yet, the vortex core and lattice in UTe_2_ have not been directly visualized and characterized. Here, by using ultralow-temperature scanning tunnelling microscopy and spectroscopy, we study the superconducting vortices on the (0−11) surface termination of UTe_2_ with an out-of-plane external magnetic field. At the centre of the vortex core, we observe a robust zero-energy vortex-core state that exhibits a cigar-shaped spatial distribution and extends to ∼30 nm along the [100] direction (crystallographic *a*-axis) of UTe_2_. Along the direction perpendicular to [100], the superconducting gap is deeper and the coherence peak on one side of the vortex core is stronger than on the opposite side, and they are even enhanced in comparison with those under zero field. Due to the anisotropy of magnetic susceptibility in UTe_2_, the asymmetric d*I*/d*V* spectra on the two sides of the vortex core result from the interplay between the magnetization-induced bound current and supercurrent around the vortex core. Our work reveals the important role of magnetization in the vortex behaviours of UTe_2_ and provides essential microscopic information for understanding its superconducting properties in a magnetic field.

## Teaser

By using an ultra-low temperature scanning tunneling microscope, researchers directly observed superconducting vortices and the unique symmetry breaking feature in the spin-triplet superconductor candidate UTe2.

## INTRODUCTION

Spin-triplet pairing is a fascinating phenomenon that has been predicted to exhibit many novel electronic properties, including fractionalized electronic states and topological edge modes [1–6]. Because of the coexistence of magnetism and superconductivity, U-based heavy-fermion superconductors are particularly promising for the realization of spin-triplet pairing [7–10]. In this context, UTe_2_ has shown strong evidence as a spin-triplet superconductor [11–20]. Although the identity of the superconducting order parameter in UTe_2_ is still under debate, several unusual superconducting properties have been reported in UTe_2_, including a large and highly anisotropic upper critical field that exceeds the Pauli limit [11–13], multiple superconducting regimes under extreme magnetic fields [13], a negligible change in the temperature-dependent nuclear magnetic resonance (NMR) shift cooling through a superconducting transition temperature (although the recent NMR measurements show a large reduction in the *a*-axis Knight shift for higher-quality UTe_2_) [15,21], the coexistence of superconductivity and ferromagnetic fluctuations from muon-spin relaxation measurements [16] and chiral in-gap states at step edges in the low-temperature scanning tunnelling microscopy and spectroscopy (STM/STS) measurements [17]. All these observations provide strong evidence in support of spin-triplet superconductivity in UTe_2_.

Superconductivity in UTe_2_ emerges upon cooling from a paramagnetic state and coexists with strong ferromagnetic fluctuations [11,16,22]. As a type-II superconductor, when a magnetic field (larger than the lower critical field but lower than the upper critical field) is applied to UTe_2_, the magnetic field penetrates into the UTe_2_ in the form of vortices that consist of both magnetic fluxes and circulating supercurrents [23]. The ferromagnetic fluctuations in the vortex of UTe_2_ can be influenced by the magnetic field within the vortex. This would result in unique vortex properties that are absent from conventional superconducting vortices. As mentioned above, UTe_2_ indeed shows several unusual behaviours in a magnetic field [11–13] and their origins still remain mysterious. Directly probing the vortices in UTe_2_ is an important step for understanding the unconventional superconducting properties of UTe_2_ in a magnetic field. The vortex core and lattice in superconductors can be directly probed by using a low-temperature and high-magnetic-field STM/STS technique [24–29]. Despite its low superconducting transition temperature and large residual density of states near zero energy [17,30–32], direct observation of the vortex core and lattice in UTe_2_ still remains elusive.

## RESULTS

**Figure 1.**
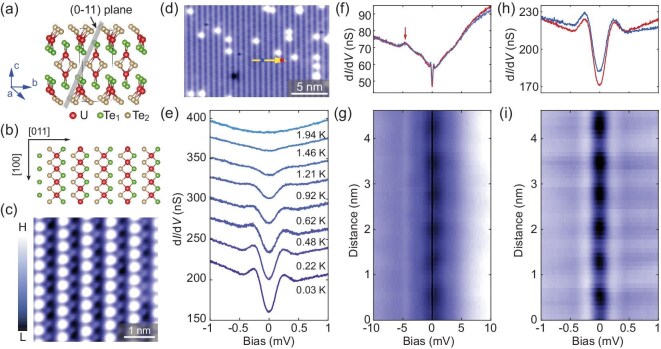
Superconductivity in UTe_2_. (a) Crystal structure of UTe_2_ with the cleavage plane shown by the grey rectangle. (b) Schematic for the structure of the (0−11) plane, which shows the Te_1_ and Te_2_ rows with the underlying U atoms. (c) High-resolution STM topography on the (0−11) surface where the Te_1_ and Te_2_ rows appear as alternating bright and dark atomic chains. (d) Typical STM topography on the (0−11) surface of UTe_2_. (e) Variable-temperature d*I*/d*V* spectra on the (0−11) surface, showing the evolution of the superconducting gap with temperature. (f, g) d*I*/d*V* line-cut profile taken along the yellow arrow in (d) with an energy range of ±10 mV (g) (set point: *V*_s_ = −10 mV, *I* = 700 pA). d*I*/d*V* spectra in (f) are taken on the bright Te chain (blue) and between the bright Te chains (red), as marked by the blue and red dots in (d). (h, i) Similarly to (f) and (g), the d*I*/d*V* spectra are taken with an energy range of ±1 mV (set point: *V*_s_ = −3 mV, *I* = 700 pA).

Here, we report an ultralow-temperature STM study of a vortex lattice and vortex-core states on the (0−11) surface of UTe_2_ single crystals. Bulk single crystals of UTe_2_ have an orthorhombic crystal structure and the superconducting transition temperature (*T*_sc_) is ∼2 K (Fig. S1). UTe_2_ single crystals typically cleave to show the (0−11) surface [17,30–33]. Similarly to the previous STM measurements, the typical STM topographies on the (0−11) surface (Fig. 1b and c) exhibit a chain-like structure in which two rows of Te atoms orient along the [100] direction (crystallographic *a*-axis) [17]. Differential tunnelling conductance (d*I*/d*V*) probes the local density of states and can measure the superconducting gap near the Fermi level. In the d*I*/d*V* spectrum taken in an energy range of ±1 meV, we observe the superconducting gap with symmetric coherence peaks located at around ±0.25 meV. The superconducting gap is gradually suppressed as the temperature increases to *T*_sc_ ∼ 2 K (Fig. 1e).

Figure 1g and i shows the line cuts of the d*I*/d*V* spectra taken in different energy ranges and along the yellow arrow in Fig. 1d. As shown in Fig. 1g, in addition to the superconducting gap at the Fermi level, there is a peak-like feature at around −4.5 mV, which may be related to the flat band derived from the *f*-electrons in UTe_2_. Although the d*I*/d*V* signal above the superconducting gap is more or less spatially uniform, the depth of the superconducting gap shows spatial dependence (Fig. 1f and h). As shown in the d*I*/d*V* line-cut profile with an energy range of ±1 meV (Fig. 1i), the depth of the superconducting gap exhibits periodic spatial modulation and the depth of the superconducting gap at the Te_2_ chain is slightly larger than that at the Te_1_ chain (Fig. 1h). Although the size of the superconducting gap is similar to that reported in previous STS measurements [17,31], the depth of the superconducting gap measured in this work is significantly larger (Fig. S2), which could be due to the lower measurement temperature (∼30 mK lattice temperature) and slightly higher *T*_sc_ for our UTe_2_ single crystal. We note that, although some properties of UTe_2_ may depend on the quality of the samples [34–38], STM is a local probe technique and the previously reported chiral edge states and charge density wave on the (0−11) surface of UTe_2_ can be repeated in our STM measurements (Fig. S3) [17,30–33].

**Figure 2.**
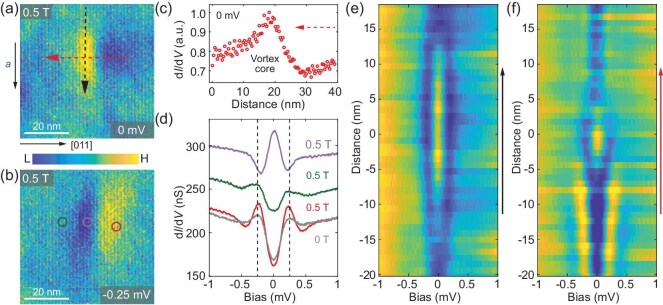
Observation of vortex and vortex-core state. (a) Zero-energy d*I*/d*V* map taken in a magnetic field *B* = 0.5 T. (b) d*I*/d*V* map taken in the same area as in (a) with 0.5 T and −0.25 mV. (c) Zero-energy d*I*/d*V* signal along the red dashed arrow in (a). (d) d*I*/d*V* spectra taken at the centre of the vortex (purple), on the left (green) and right (red) sides of the vortex core with 0.5 T, and the positions for these spectra are marked by the coloured circles in (b). The spectra are vertically offset for clarity. The grey d*I*/d*V* spectrum taken with a zero magnetic field is shown for comparison. (e, f) d*I*/d*V* line-cut profiles taken along the (e) black and (f) red dashed arrows in (a), which shows the evolution of the zero-energy vortex-core state inside the superconducting gap. The d*I*/d*V* maps and d*I*/d*V* spectra in this figure are taken with set point *V*_s_ = −3 mV and *I* = 700 pA.

Having confirmed the zero-field superconductivity in UTe_2_, we next investigate the vortex lattice and vortex-core states by performing d*I*/d*V* measurements with an external magnetic field perpendicular to the (0–11) surface. In this case, the magnetic field is along the direction with an angle offset ∼24° from the crystallographic *b*- to *c*-axes [39]. The spatial distribution of the vortex core reflects the quasiparticle wave function and can be mapped out by performing d*I*/d*V* map measurements. Figure 2a shows the zero-energy d*I*/d*V* map and a cigar-shaped vortex core appears elongated along the [100] direction. Figure 2b is the d*I*/d*V* map taken at the coherence peak energy (–0.25 mV) and in the same area as that shown in Fig. 2a. In the d*I*/d*V* spectrum taken at the centre of the vortex, we observe a zero-energy vortex-core state with the full width at half maximum ∼0.2 mV (Fig. 2d). Figure 2e and f shows the d*I*/d*V* line-cut spectra taken along the [100] and [011] directions, respectively (denoted by the dashed arrows in Fig. 2a). In the line cut of the d*I*/d*V* spectra along the [011] direction, the zero-energy conductance peak is located within a narrow spatial range (Fig. 2f). However, the zero-energy peak extends to ∼30 nm along the [100] direction and does not split (Fig. 2e). This indicates that the zero-energy vortex-core state is highly anisotropic, which can be explained by the anisotropy of the Ginzburg–Landau coherence length *ξ* along the two directions. The cigar-shaped vortex should be attributed to the anisotropic Fermi surface and the superconducting gap structure in UTe_2_ [25]. By fitting the zero-energy conductance values as a function of the position with an exponential decay, the extracted coherence lengths along the two directions are *ξ*_1_∼ 15 nm and *ξ*_2_∼ 5 nm, respectively (Fig. S4).

Another prominent feature shown in the d*I*/d*V* maps is that the d*I*/d*V* signal on the right side of the vortex core (along the [011] direction from the vortex centre) appears different from that on its left side (Fig. 2a–c). The zero-energy d*I*/d*V* signal on the right side of the vortex core is significantly lower than that on the left side (Fig. 2a and c) and the d*I*/d*V* signal at the coherence peak energy (–0.25 mV) has higher intensity on the right side (Fig. 2b). This indicates that the right side of the vortex shows a deeper superconducting gap and a stronger coherence peak, which can be clearly seen in the d*I*/d*V* spectra taken on the two sides of the vortex core (Fig. 2d). More surprisingly, the right-side superconducting gap and the coherence peak are even more prominent than those taken in a zero magnetic field (Fig. 2d). Enhancement of the superconductivity appears within an area of ∼20 × 20 nm^2^ on the right side of the vortex core and it induces inversion symmetry breaking along the [011] direction. To exclude the possibility that this inversion symmetry breaking near the vortex core is induced by local defects, we perform line cuts of the d*I*/d*V* spectra measurement without a magnetic field along the same red dashed arrow in Fig. 2a and no symmetry-breaking feature is observed (Fig. S5). We also note that the symmetry-breaking feature only appears in the d*I*/d*V* spectra within the superconducting gap energy range (Fig. S6).

**Figure 3.**
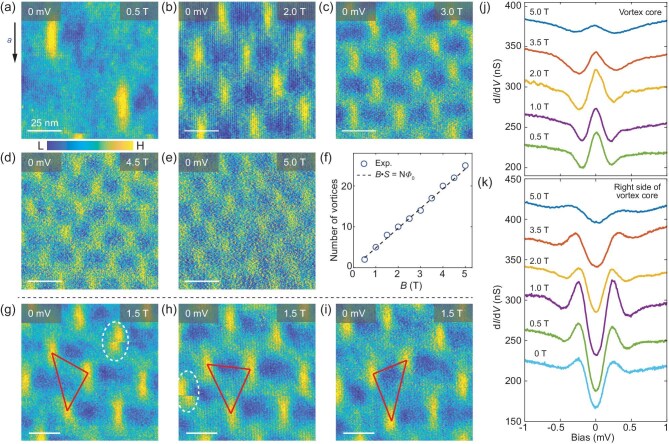
Evolution of vortex lattice as a function of external magnetic field. (a–e) Zero-energy d*I*/d*V* maps taken at magnetic fields of (a) 0.5, (b) 2.0, (c) 3.0, (d) 4.5 and (e) 5.0 T. (f) Number of vortices (blue circles) as a function of magnetic fields perpendicular to the surface. The dashed line represents the theoretical values of the number of vortices in an area of 100 × 100 nm^2^. (g–i) Series of zero-energy d*I*/d*V* maps taken with magnetic fields of 1.5 T on the same area within 48 hours. The red triangles indicate the relative positions of the vortex cores. The dashed ellipses mark the moved vortex cores during the d*I*/d*V* map measurements. Scale bar in (a–i): 25 nm. (j) Magnetic-field dependence of the d*I*/d*V* spectra taken at the centre of the vortex. (k) Magnetic-field dependence of the d*I*/d*V* spectra taken on the right side of the vortex and the d*I*/d*V* spectrum taken with a zero magnetic field. The spectra in (j) and (k) are vertically offset for clarity. The d*I*/d*V* maps and d*I*/d*V* spectra in this figure are taken with set point *V*_s_ = −3 mV and *I* = 700 pA.

To reveal the evolution of the symmetry breaking near the vortex core with external magnetic fields, we perform the magnetic-field-dependent measurements for the vortices in UTe_2_. Figure 3a–e shows the zero-energy d*I*/d*V* maps taken with different magnetic fields perpendicular to the (0−11) surface and the density of the vortices is proportional to the strength of the magnetic field. Figure 3f shows the number of vortices as a function of the external magnetic fields in an area of 100 × 100 nm^2^, which indicates that each vortex carries one magnetic flux quanta (*ϕ*_0_ ∼ 2.07 × 10^−15^ Wb). In low magnetic fields, such as 0.5 T, the symmetry breaking near each vortex core can be clearly seen (Fig. 3a). As the magnetic fields increases to above ∼2 T, the vortices form a triangular lattice and the right side of a vortex often overlaps with the left side of the neighbouring vortex. This makes the asymmetry near the vortex cores difficult to distinguish (Fig. 3b–e). As the magnetic field increases to ∼2 T, the depth of the superconducting gap in the d*I*/d*V* spectra measured on the right side of the vortex gradually gets smaller (Fig. 3k). At the same time, the intensity of the zero-energy vortex-core state decreases, which could be due to the vortex–vortex interaction (Fig. 3j). Similar behaviour has also been observed for the zero-energy vortex-core state in an iron-based superconductor (LiFeAs) [40].

**Figure 4.**
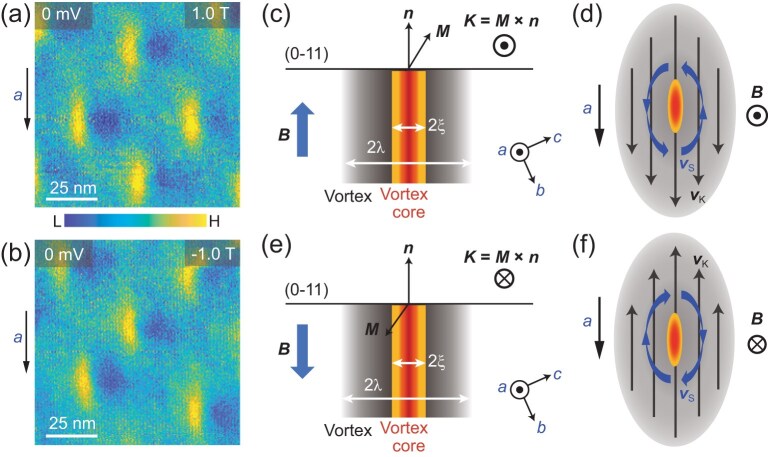
Magnetic-field-direction independence and phenomenology of the symmetry breaking near the vortex core. (a, b) Zero-energy d*I*/d*V* maps taken in the same area with magnetic fields of (a) 1 and (b) −1 T (set point: *V*_s_ = −3 mV, *I* = 700 pA). (c) Schematic showing the magnetization (***M***) with a magnetic field along the surface normal vector (***n***) and the bound current (***K = M × n***). *ξ* denotes the coherence length and *λ* is the London penetration depth in UTe_2_. (d) Schematic showing the bound current (***K = M × n***) with superfluid velocity (***v*_K_**) and the circulating supercurrent with superfluid velocity (***v*_s_**) around the vortex core. The red–orange and grey ovals represent the vortex core and the vortex in UTe_2_, respectively. (e, f) Similar to (c) and (d), but with the magnetic field along the opposite direction.

During the d*I*/d*V* map measurements, we find that the vortices in UTe_2_ are weakly pinned and easy to move. Figure 3g–i shows three typical zero-energy d*I*/d*V* maps taken in the same area and within 48 hours. We can see that, although the vortices can be stable for a few hours to allow the d*I*/d*V* map measurements, the vortex lattice keeps changing for a longer time. The dashed ellipses shown in Fig. 3g and h mark the moved vortex cores captured in the d*I*/d*V* map measurements and the vortex motion results in a break on a certain line of the d*I*/d*V* map. Interestingly, no matter where the vortices are located, the symmetry breaking is associated with all the vortex cores. This also rules out the possibility that the symmetry breaking is due to the local defects near the vortex core. The weakly pinning effect of the vortices in UTe_2_ is consistent with the recent direct current resistivity measurements [41]. Furthermore, we find that this kind of symmetry breaking near the vortex core is independent of the direction of the out-of-plane magnetic field. Figure 4a and b shows the zero-energy d*I*/d*V* maps taken on the same area with magnetic fields of +1 and −1 T, and they show the same asymmetry. This demonstrates that the symmetry breaking near the elongated vortex core is not due to the possible misalignment between the direction of the magnetic field and the surface normal. Otherwise, when the out-of-plane direction of the magnetic field is reversed, the asymmetry near the vortex core should also reverse.

## DISCUSSION

Finally, we discuss the possible origin of the symmetry breaking near the vortex core of UTe_2_. First, the maximum magnetic field that we applied is ∼5 T and perpendicular to the (0−11) surface. It is above the Pauli limit for UTe_2_ (∼3.7 T for *T*_sc_ ∼ 2 K), but below the strength of the magnetic field for inducing the re-entrant superconducting phases [13], which indicates that the STM measurements in this work are within a single superconducting phase of UTe_2_ (SC1 phase). The SC1 phase in UTe_2_ emerges upon cooling from a nearly ferromagnetic state with the crystallographic *b*-axis as the magnetic hard axis [11,42]. When an out-of-plane external magnetic field is applied, the magnetic fields enter in the form of vortices consisting of magnetic fluxes and a circulating supercurrent. The STM measures the vortex-core states that are distributed on the length scale of the coherence length (*ξ*). The magnetic fields decay from the vortex core on the length scale of the London penetration depth (*λ*) (Fig. 4c and e) [23]. For the U-based superconductors, the London penetration depth can be several hundreds of nanometres or even larger [43].

Around the vortex-core region, the magnetic moments from the U *f*-electrons can be polarized by the magnetic fields within the vortex, which induces magnetization (***M***). Since the *b*-axis is the magnetic hard axis of UTe_2_, when the magnetic field is applied perpendicular to the (0−11) surface, the magnetization would rotate toward the *c*-axis from the magnetic field direction (Fig. 4c and e). This would induce a surface-bound current (***K***) that is calculated by using ***M × n*** (where ***n*** is the surface normal vector). In this situation, on the left side of the vortex core, the direction of the bound current is along the same direction of the supercurrent (Fig. 4d). However, on the right side of the vortex, their flow directions are opposite (Fig. 4d). This phenomenon would result in a smaller net supercurrent on the right side of the vortex core than on the left side. The following question is: how does this induce the asymmetric superconducting quasiparticle spectrum?

Due to the Doppler shift, the current in the superconductors weakens pair correlations and shifts the energy of the Bogoliubov quasiparticle excitations [44,45]. In our case, one possible scenario is that the superfluid velocity on the right side of the vortex core (***v*_R_**=***v*_s_ – *v*_K_**) is smaller than that on the left side (***v*_L_** =***v*_s_**+ ***v*_K_**), where ***v*_s_** and ***v*_K_** are the superfluid velocities related to the supercurrent and surface-bound current, respectively. Therefore, the Doppler shift effect on the right side of the vortex core is weaker than on the left side, which gives rise to the asymmetric d*I*/d*V* spectra. When the direction of the out-of-plane magnetic fields is reversed, the magnetization direction within the vortex also changes (Fig. 4e and f). In this case, both the direction of the bound current and the circulation direction of the supercurrent reverse, which keeps the asymmetry unchanged. This is also consistent with the magnetic-field-direction-dependent d*I*/d*V* maps shown in Fig. 4a and b.

The coexistence of magnetization and the superconducting gap near the vortex core supports the spin-triplet superconductivity in UTe_2_. General symmetry analysis for the odd-parity spin-triplet superconducting gap function of UTe_2_ can be divided into two classes: chiral and non-chiral [46]. So far, the experimental identification of the superconducting gap symmetry of UTe_2_ remains still under debate [17,18,21,47,48]. It can be regarded that the spatial configuration around a superconducting vortex, separating the superconducting bulk from the non-superconducting vortex core, is topologically equivalent to a superconductor with left and right boundaries. The symmetry breaking observed here is analogous to the previously reported chiral boundary states at the step edges of UTe_2_ [17] and our proposed explanation for the magnetization-induced asymmetry around the vortex core indicates that the magnetic properties at the step edges of UTe_2_ may play an important role in the chiral boundary modes in UTe_2_.

## CONCLUSION

Our STM data reveal many intriguing features for the vortex-core states and vortex lattice in UTe_2_. The elongation behaviour of the robust zero-energy vortex-core state should be a combined effect of the Fermi surface anisotropy and the superconducting gap structure of UTe_2_. However, whether this zero-energy vortex-core state is the Majorana zero mode in a spin-triplet superconductor needs further experimental investigation. We detect the asymmetric d*I*/d*V* spectra on the two sides of the elongated vortex core, which are induced by the magnetization-induced bound current. Our findings also provide a new clue for understanding the chiral boundary modes in UTe_2_. The enhanced depth of the superconducting gap and coherence peak on one side of the vortex core is extremely special and further theoretical modelling is needed to reveal its origins and implications. We also expect that this kind of symmetry breaking in the vortex should be a general phenomenon for superconductors with strong and anisotropic paramagnetism.

*Note added.* After submitting the manuscript, we became aware of the other two STM studies about the superconducting vortices in UTe_2_ [49,50].

## METHODS

### Single-crystal growth

High-quality UTe_2_ crystals were successfully synthesized by using the molten salt flux method [51]. Prior to the preparation, natural uranium metal (3 N) was polished by using an electric sander to eliminate surface oxides and then cleaned with alcohol and acetone. High-purity NaCl (4 N) and KCl (4 N) were finely ground and then baked in an oven at 120°C for 24 hours to remove moisture. Initially, precise amounts of high-purity uranium, tellurium powder (5 N), NaCl and KCl were weighed and mixed at a molar ratio of 1:1.71:20:20. This mixture was placed into a small Al_2_O_3_ crucible and subsequently loaded into a 13-cm-long quartz ampoule with an inner diameter of 20 mm, which was then sealed under a pressure of ∼10^−3^ Pa. The sealed ampoule was introduced into a shaft furnace, in which the temperature was gradually increased to 950°C over 24 hours and maintained for another 24 hours. Afterwards, the temperature was adjusted to 650°C over a period of 2–3 weeks to promote crystal growth. After cooling to room temperature, the content in the Al_2_O_3_ crucible was soaked in deionized water for 24 hours to remove NaCl and KCl, resulting in the successful production of the rod-like UTe_2_ single crystals (Fig. S1).

### STM/STS measurements

STM experiments were conducted by using an ultralow-temperature STM system at a base temperature of 30 mK with an effective electronic temperature of ∼200 mK (Unisoku 1600) [52]. STM measurements were performed by using chemically etched tungsten tips. The tungsten tips were flashed by using electron-beam bombardment for 2 minutes before use. The UTe_2_ single-crystal sample was cleaved at 77 K and then transferred immediately into the STM head for measurement. The d*I*/d*V* spectra were acquired by using a standard lock-in technique at a modulation frequency of 910 Hz.

## Supplementary Material

nwaf267_Supplemental_File

## References

[1] Mackenzie AP, Maeno Y. The superconductivity of Sr_2_RuO_4_ and the physics of spin-triplet pairing. Rev Mod Phys 2003; 75: 657–712. 10.1103/RevModPhys.75.657

[2] Read N, Green D. Paired states of fermions in two dimensions with breaking of parity and time-reversal symmetries and the fractional quantum Hall effect. Phys Rev B 2000; 61: 10267–97. 10.1103/PhysRevB.61.10267

[3] Vakaryuk V, Leggett AJ. Spin polarization of half-quantum vortex in systems with equal spin pairing. Phys Rev Lett 2009; 103: 057003. 10.1103/PhysRevLett.103.057003 19792527

[4] Hsieh TH, Fu L. Majorana fermions and exotic surface andreev bound states in topological superconductors: application to Cu_x_B_2_Se_3_. Phys Rev Lett 2012; 108: 107005. 10.1103/PhysRevLett.108.107005 22463445

[5] Salomaa MM, Volovik GE. Quantized vortices in superfluid ^3^He. Rev Mod Phys 1987; 59: 533–613. 10.1103/RevModPhys.59.533 9935414

[6] Tsutsumi Y, Machida K. Topological spin texture and *d*-vector rotation in spin-triplet superconductors: a case of UTe_2_. Phys Rev B 2024; 110: L060507. 10.1103/PhysRevB.110.L060507

[7] Stewart GR, Fisk Z, Willis JO et al. Possibility of coexistence of bulk superconductivity and spin fluctuations in UPt_3_. Phys Rev Lett 1984; 52: 679–82. 10.1103/PhysRevLett.52.679

[8] Saxena SS, Agarwal P, Ahilan K et al. Superconductivity on the border of itinerant-electron ferromagnetism in UGe_2_. Nature 2000; 406: 587–92. 10.1038/35020500 10949292

[9] Aoki D, Huxley A, Ressouche E et al. Coexistence of superconductivity and ferromagnetism in URhGe. Nature 2001; 413: 613–6. 10.1038/35098048 11595943

[10] Huy NT, Gasparini A, de Nijs DE et al. Superconductivity on the border of weak itinerant ferromagnetism in UCoGe. Phys Rev Lett 2007; 99: 067006. 10.1103/PhysRevLett.99.067006 17930860

[11] Ran S, Eckberg C, Ding Q-P et al. Nearly ferromagnetic spin-triplet superconductivity. Science 2019; 365: 684–7. 10.1126/science.aav8645 31416960

[12] Aoki D, Nakamura A, Honda F et al. Unconventional superconductivity in heavy fermion UTe_2_. J Phys Soc Jpn 2019; 88: 043702. 10.7566/JPSJ.88.043702

[13] Ran S, Liu IL, Eo YS et al. Extreme magnetic field-boosted superconductivity. Nat Phys 2019; 15: 1250–4. 10.1038/s41567-019-0670-x PMC8201648 34131432

[14] Aoki D, Brison J-P, Flouquet J et al. Unconventional superconductivity in UTe_2_. J Phys Condens Matter 2022; 34: 243002. 10.1088/1361-648X/ac5863 35203074

[15] Nakamine G, Kinjo K, Kitagawa S et al. Anisotropic response of spin susceptibility in the superconducting state of UTe_2_ probed with ^125^Te-NMR measurement. Phys Rev B 2021; 103: L100503. 10.1103/PhysRevB.103.L100503

[16] Sundar S, Gheidi S, Akintola K et al. Coexistence of ferromagnetic fluctuations and superconductivity in the actinide superconductor UTe_2_. Phys Rev B 2019; 100: 140502(R). 10.1103/PhysRevB.100.140502 PMC8201662 34131607

[17] Jiao L, Howard S, Ran S et al. Chiral superconductivity in heavy-fermion metal UTe_2_. Nature 2020; 579: 523–7. 10.1038/s41586-020-2122-2 32214254

[18] Hayes IM, Wei DS, Metz T et al. Multicomponent superconducting order parameter in UTe_2_. Science 2021; 373: 797–801. 10.1126/science.abb0272 34385397

[19] Bae S, Kim H, Eo YS et al. Anomalous normal fluid response in a chiral superconductor UTe_2_. Nat Commun 2021; 12: 2644. 10.1038/s41467-021-22906-6 33976162 PMC8113495

[20] Ishihara K, Roppongi M, Kobayashi M et al. Chiral superconductivity in UTe_2_ probed by anisotropic low-energy excitations. Nat Commun 2023; 14: 2966. 37221184 10.1038/s41467-023-38688-y PMC10205722

[21] Matsumura H, Fujibayashi H, Kinjo K et al. Large reduction in the a-axis Knight shift on UTe_2_ with *T*c = 2.1 K. J Phys Soc Jpn 2023; 92: 063701. 10.7566/JPSJ.92.063701

[22] Lewin SK, Frank CE, Ran S et al. A review of UTe_2_ at high magnetic fields. Rep Prog Phys 2023; 86: 114501. 10.1088/1361-6633/acfb93 37729901

[23] Abrikosov AA . The magnetic properties of superconducting alloys. J Phys Chem Solids 1957; 2: 199–208. 10.1016/0022-3697(57)90083-5

[24] Hess HF, Robinson RB, Dynes RC et al. Scanning-tunneling-microscope observation of the Abrikosov flux lattice and the density of states near and inside a fluxoid. Phys Rev Lett 1989; 62: 214–6. 10.1103/PhysRevLett.62.214 10039952

[25] Hess HF, Robinson RB, Waszczak JV. Vortex-core structure observed with a scanning tunneling microscope. Phys Rev Lett 1990; 64: 2711–4. 10.1103/PhysRevLett.64.2711 10041790

[26] Song C-L, Wang Y-L, Cheng P et al. Direct observation of nodes and twofold symmetry in FeSe superconductor. Science 2011; 332: 1410–3. 10.1126/science.1202226 21680839

[27] Suderow H, Guillamón I, Rodrigo JG et al. Imaging superconducting vortex cores and lattices with a scanning tunneling microscope. Supercond Sci Technol 2014; 27: 063001. 10.1088/0953-2048/27/6/063001

[28] Wang D, Kong L, Fan P et al. Evidence for Majorana bound states in an iron-based superconductor. Science 2018; 362: 333–5. 10.1126/science.aao1797 30115743

[29] Chen M, Chen X, Yang H et al. Discrete energy levels of Caroli-de Gennes-Matricon states in quantum limit in FeTe_0.55_Se_0.45_. Nat Commun 2018; 9: 970. 10.1038/s41467-018-03404-8 29511191 PMC5840178

[30] Aishwarya A, May-Mann J, Raghavan A et al. Magnetic-field-sensitive charge density waves in the superconductor UTe_2_. Nature 2023; 618: 928–33. 10.1038/s41586-023-06005-8 37380690

[31] Gu Q, Carroll JP, Wang S et al. Detection of a pair density wave state in UTe_2_. Nature 2023; 618: 921–7. 10.1038/s41586-023-05919-7 37380691 PMC10307636

[32] Aishwarya A, May-Mann J, Almoalem A et al. Melting of the charge density wave by generation of pairs of topological defects in UTe_2_. Nat Phys 2024; 20: 964–9. 10.1038/s41567-024-02429-9

[33] LaFleur A, Li H, Frank CE et al. Inhomogeneous high temperature melting and decoupling of charge density waves in spin-triplet superconductor UTe_2_. Nat Commun 2024; 15: 4456. 10.1038/s41467-024-48844-7 38796494 PMC11127989

[34] Ajeesh MO, Bordelon M, Girod C et al. Fate of time-reversal symmetry breaking in UTe_2_. Phys Rev X 2023; 13: 041019. 10.1103/PhysRevX.13.041019

[35] Wei DS, Saykin D, Miller OY et al. Interplay between magnetism and superconductivity in UTe_2_. Phys Rev B 2022; 105: 024521. 10.1103/PhysRevB.105.024521

[36] Rosa PFS, Weiland A, Fender SS et al. Single thermodynamic transition at 2 K in superconducting UTe_2_ single crystals. Commun Mater 2022; 3: 33. 10.1038/s43246-022-00254-2

[37] Thomas SM, Stevens C, Santos FB et al. Spatially inhomogeneous superconductivity in UTe_2_. Phys Rev B 2021; 104: 224501. 10.1103/PhysRevB.104.224501

[38] Azari N, Yakovlev M, Rye N et al. Absence of spontaneous magnetic fields due to time-reversal symmetry breaking in bulk superconducting UTe_2_. Phys Rev Lett 2023; 131: 226504. 10.1103/PhysRevLett.131.226504 38101387

[39] Aoki D, Sheikin I, Marquardt N et al. High field superconducting phases of ultra clean single crystal UTe_2_. J Phys Soc Jpn 2024; 93: 123702. 10.7566/JPSJ.93.123702

[40] Li M, Li G, Cao L et al. Ordered and tunable Majorana-zero-mode lattice in naturally strained LiFeAs. Nature 2022; 606: 890–5. 10.1038/s41586-022-04744-8 35676489

[41] Tokiwa Y, Sakai H, Kambe S et al. Anomalous vortex dynamics in the spin-triplet superconductor UTe_2_. Phys Rev B 2023; 108: 144502. 10.1103/PhysRevB.108.144502

[42] Rosuel A, Marcenat C, Knebel G et al. Field-induced tuning of the pairing state in a superconductor. Phys Rev X 2023; 13: 011022. 10.1103/PhysRevX.13.011022

[43] Gross F, Andres K, Chandrasekhar BS. Experimental determination of the absolute value of the London penetration depth in the heavy fermion superconductors UBe_13_ und UPt_3_. Physica C 1989; 162–164: 419–20. 10.1016/0921-4534(89)91084-8

[44] Zhu Z, Papaj M, Nie X-A et al. Discovery of segmented Fermi surface induced by Cooper pair momentum. Science 2021; 374: 1381–5. 10.1126/science.abf1077 34709939

[45] Liu X, Chong YX, Sharma R et al. Atomic-scale visualization of electronic fluid flow. Nat Mater 2021; 20: 1480–4. 10.1038/s41563-021-01077-1 34462570

[46] Kallin C, Berlinsky J. Chiral superconductors. Rep Prog Phys 2016; 79: 054502. 10.1088/0034-4885/79/5/054502 27088452

[47] Iguchi Y, Man H, Thomas SM et al. Microscopic imaging homogeneous and single phase superfluid density in UTe_2_. Phys Rev Lett 2023; 130: 196003. 10.1103/PhysRevLett.130.196003 37243629

[48] Suetsugu S, Shimomura M, Kamimura M et al. Fully gapped pairing state in spin-triplet superconductor UTe_2_. Sci Adv 2024; 10: eadk3772. 10.1126/sciadv.adk3772 38324692 PMC10849587

[49] Sharma N, Toole M, McKenzie J et al. Observation of persistent zero modes and superconducting vortex doublets in UTe_2_. arXiv: 2503.17450. 10.1021/acsnano.5c08406 40856047

[50] Yin R, Li Y, Du Z et al. Yin-Yang vortex on UTe_2_ (011) surface. arXiv: 2503.21506. 10.1038/s41467-026-72162-9 PMC13276238 42000710

[51] Sakai H, Opletal P, Tokiwa Y et al. Single crystal growth of superconducting UTe_2_ by molten salt flux method. Phys Rev Mater 2022; 6: 073401. 10.1103/PhysRevMaterials.6.073401

[52] Zhong R, Yang Z, Wang Q et al. Spatially dependent in-gap states induced by Andreev tunneling through a single electronic state. Nano Lett 2024; 24: 8580–6. 10.1021/acs.nanolett.4c01581 38967330

