# Supplementary material for: Spectroscopic evidence of symmetry breaking in the superconducting vortices of UTe2
Source: Natl Sci Rev. 2025 Jul 4;12(8):nwaf267. doi: 10.1093/nsr/nwaf267 (PMC12365754; doi:10.1093/nsr/nwaf267)
Supplement: nwaf267_Supplemental_File [file nwaf267_supplemental_file.docx]

**Supplemental Material**

**Spectroscopic evidence of symmetry breaking in the superconducting vortices of UTe_2_**

Zhongzheng Yang^1,*^, Fanbang Zheng^1,*^, Dingsong Wu^2,*^, Bin-Bin Zhang^3,*^, Ning Li^3^, Wenhui Li^1,4^, Chaofan Zhang^3,†^, Guang-Ming Zhang^1^, Xi Chen^5^, Yulin Chen^1,2,4,†^, Shichao Yan^1,4,†^

*^1^State Key Laboratory of Quantum Functional Materials, School of Physical Science and Technology, ShanghaiTech University, Shanghai, China*

*^2^Department of Physics, University of Oxford, Oxford, UK*

*^3^Nanhu Laser Laboratory, Changsha, China*

*^4^ShanghaiTech Laboratory for Topological Physics, ShanghaiTech University, Shanghai, China*

*^5^State Key Laboratory of Low-Dimensional Quantum Physics, Department of Physics, Tsinghua University, Beijing, China*

*^*^These authors contributed equally*

^†^*Email: yanshch@shanghaitech.edu.cn; yulin.chen@physics.ox.ac.uk; hjroland@163.com*

**Table of contents**

**1. High quality of UTe_2_ single crystal**

**2. Comparison with the previously reported d*I*/d*V* spectra**

**3. Charge density wave and chiral edge states on the (0−11) surface of UTe_2_**

**4. Decay lengths of the zero-energy d*I*/d*V* signal in Figures 2e and 2f**

**5. Linecuts of d*I*/d*V* spectra taken with and without external magnetic fields**

**6. Linecuts of d*I*/d*V* spectra taken with different energy ranges**

1. **High quality of UTe_2_ single crystal**


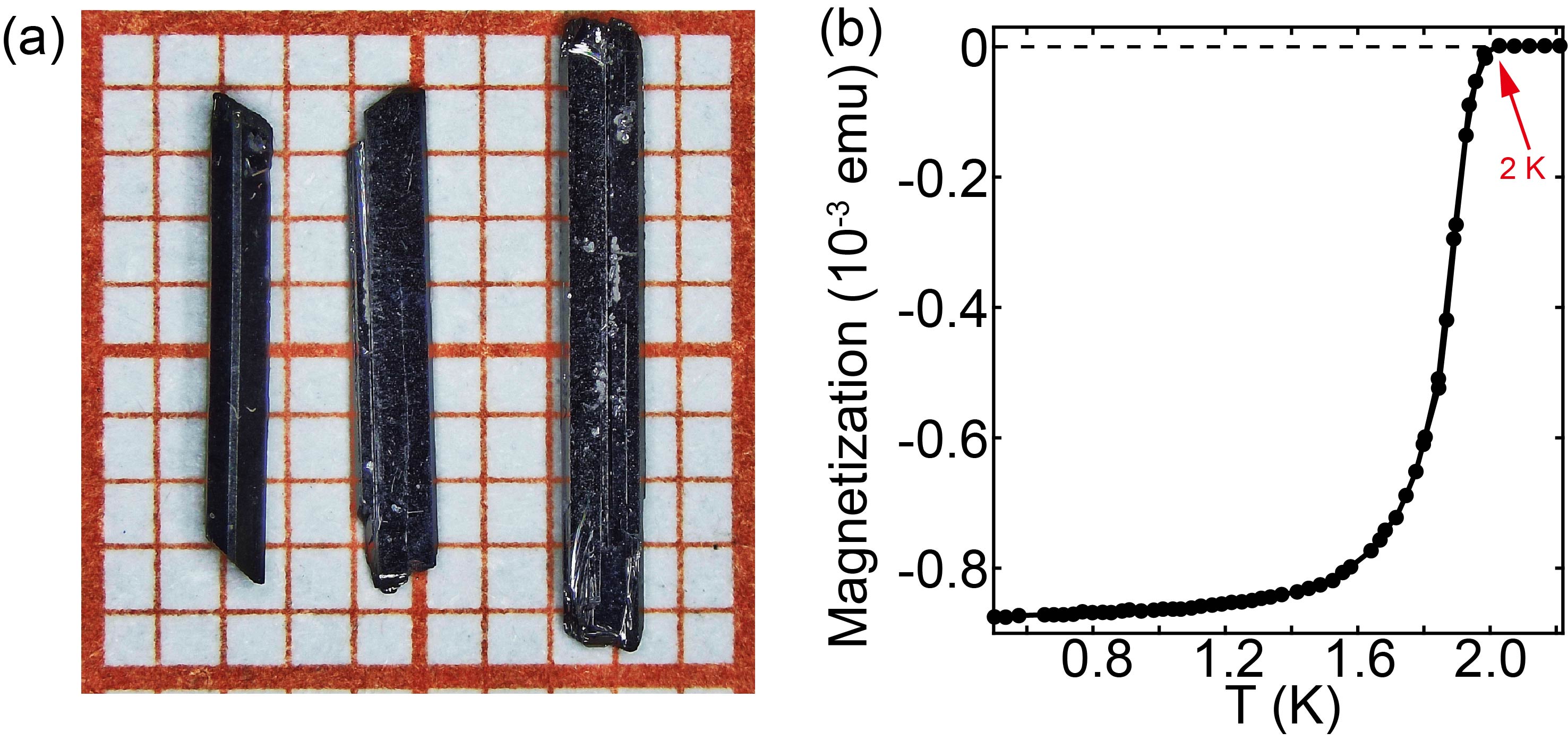


FIG. S1. (a) Optical image of the as-grown UTe_2_ single crystals. (b) The typical temperature dependence of magnetic susceptibility at 10 Oe for UTe_2_ with the superconducting transition temperature ~2 K.

1. **Comparison with the previously reported d*I*/d*V* spectra**

There have been two STM works (Refs. [1, 2]) which show the superconducting gap of UTe_2_ at ~300 mK temperature. We compare the d*I*/d*V* spectra at ~300 mK in Refs. [1, 2] with the typical d*I*/d*V* spectrum measured at ~30 mK in our work. We can see that the depth of the superconducting gap measured at ~30 mK is about 3 times larger than that measured at ~300 mK [Fig. S2(a)]. As shown in Fig.S2(b), our 480 mK d*I*/d*V* spectrum is comparable with the previously reported d*I*/d*V* spectra at ~300 mK.


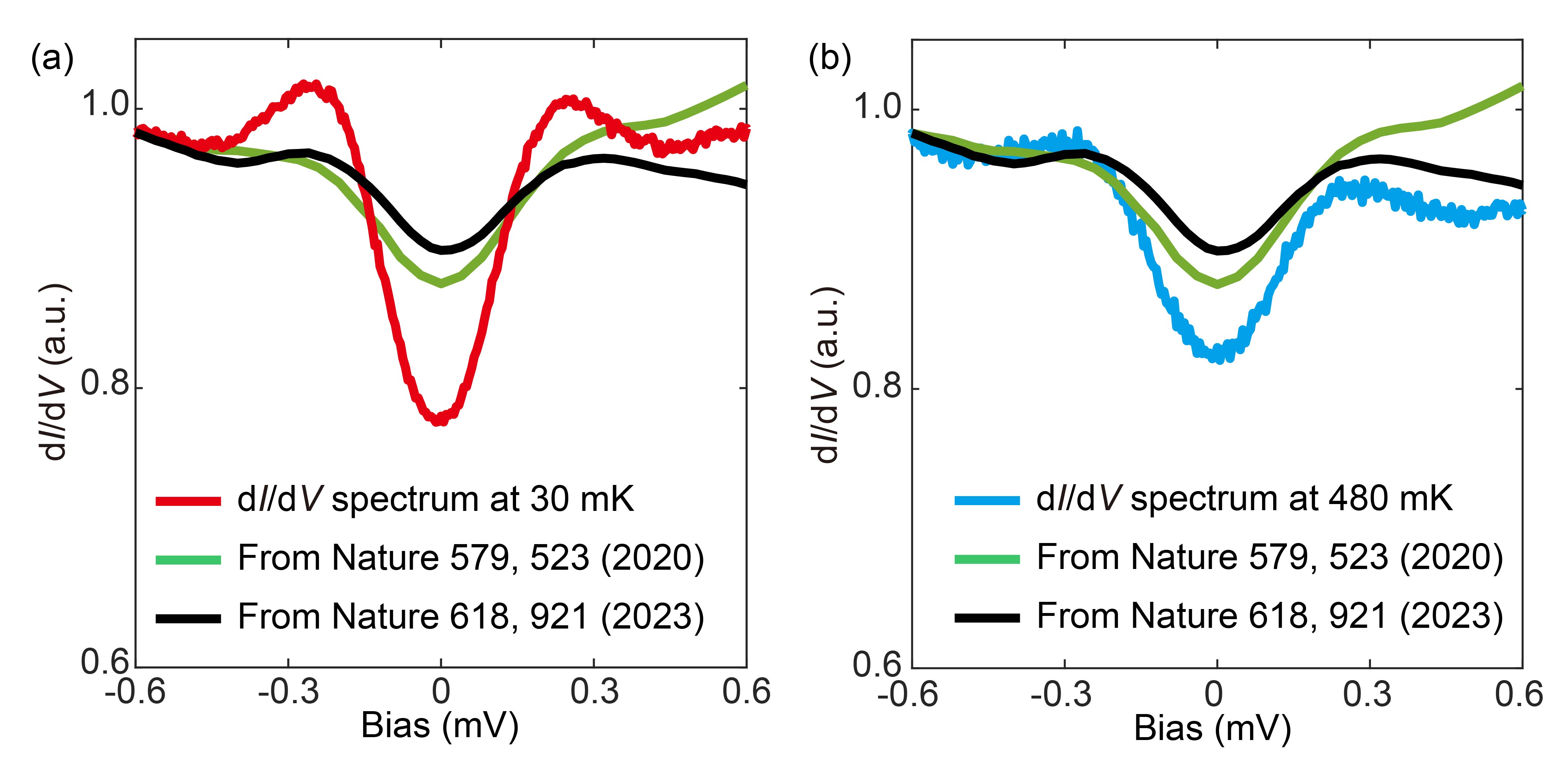


FIG. S2. Comparison between the d*I*/d*V* spectra measured at 30 mK (a) and 480 mK (b) in this work and the previously reported d*I*/d*V* spectra at ~300 mK (green and black).

1. **Charge density wave state and chiral edge states on the (0−11) surface of UTe_2_**

Charge density wave and chiral edge states have been reported in the previously published STM works on the (0−11) surface of UTe_2_ [1-5]. In our STM measurements, these features can also be observed (Fig. S3). We also find that the peak-dip feature in the chiral edge states can be reduced by applying external magnetic fields [Figs. S3(f) and S3(g)].


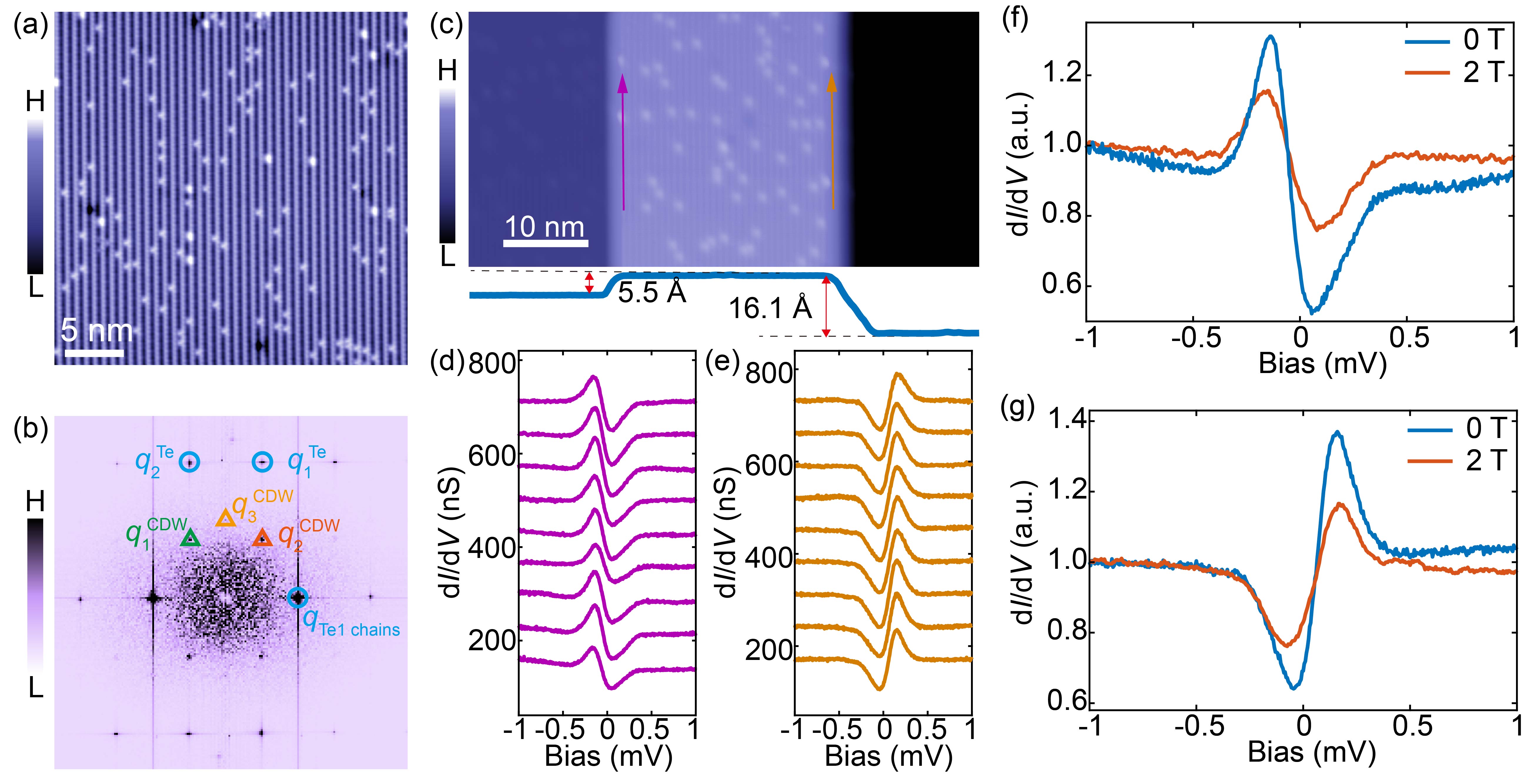


FIG. S3. (a) Typical STM topography of UTe_2_. (b) Fourier transform (FT) image of the STM topography in **a** with the lattice Bragg peaks and charge density wave peaks marked. The FT image is similar to that reported in *Nature Physics* 20, 964 (2024). (c) STM topography of UTe_2_ with terraces. (d),(e) Linecuts of d*I*/d*V* spectra taken along the purple and orange arrows in (c), respectively, which show the chiral in-gap states in UTe_2_. (f),(g) Two different kinds of chiral edge states under 0 T (blue) and 2 T (orange) external magnetic fields.

1. **Decay lengths of the zero-energy d*I*/d*V* signal in Figures 2(e) and 2(f)**


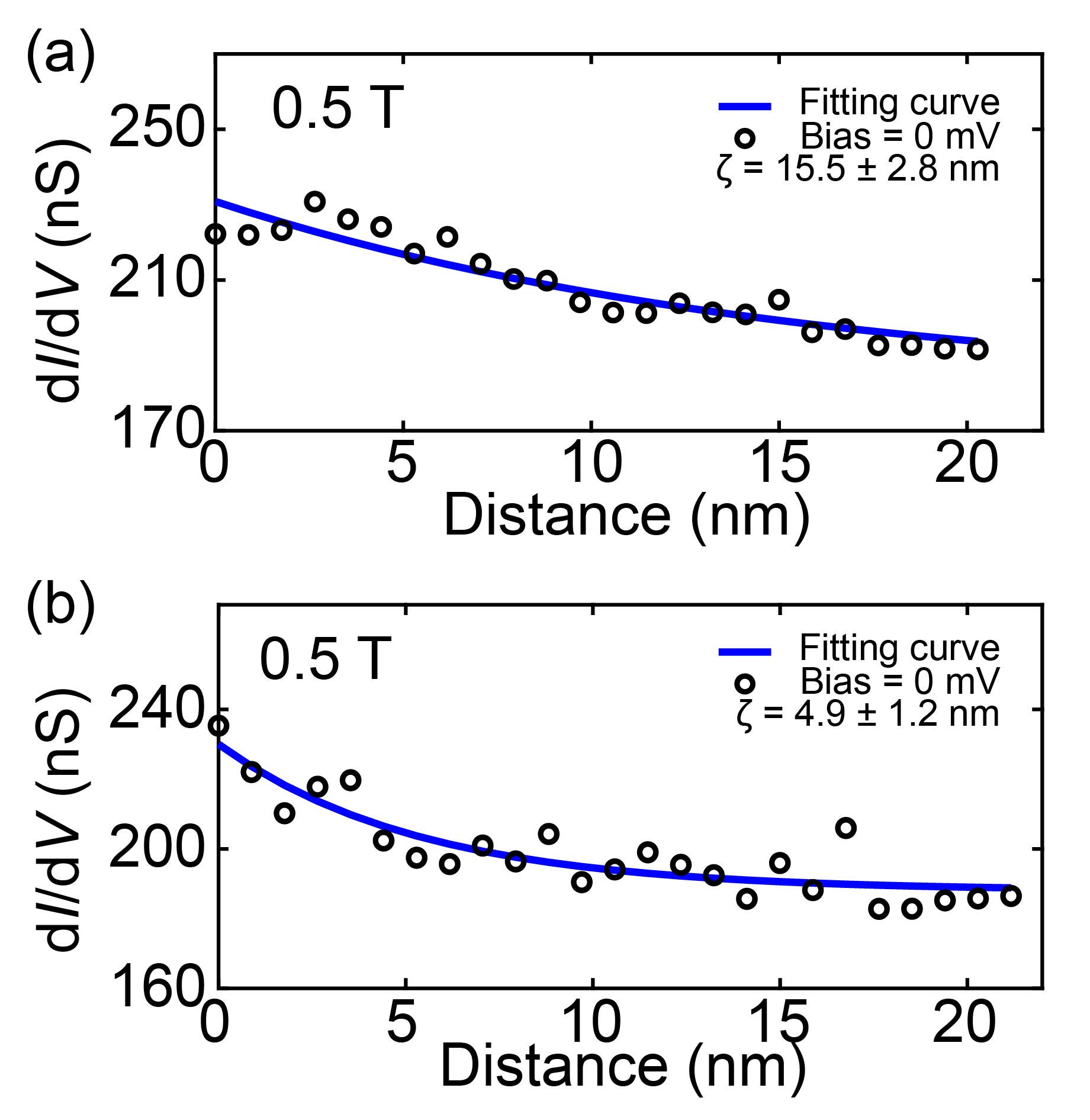


FIG. S4. (a),(b) Spatial dependence of the zero-energy d*I*/d*V* signal from the center of the vortex shown in Figs. 2(e) and 2(f), respectively. ξ_1_ and ξ_2_ denote the characteristic decay lengths obtained from the exponential fits (blue curves). The zero-energy d*I*/d*V* signal in (b) is extracted from the center of the vortex to the left side of the vortex core.

1. **Linecuts of d*I*/d*V* spectra taken with and without external magnetic fields**

In order to exclude the possibility that the local symmetry breaking is induced by the defects on the right side of the vortex core in UTe_2_. We perform linecuts of d*I*/d*V* spectra taken along the same dashed lines for Figs. 2(e) and 2(f) with zero magnetic field, where there is no symmetry breaking feature (Fig. S5).


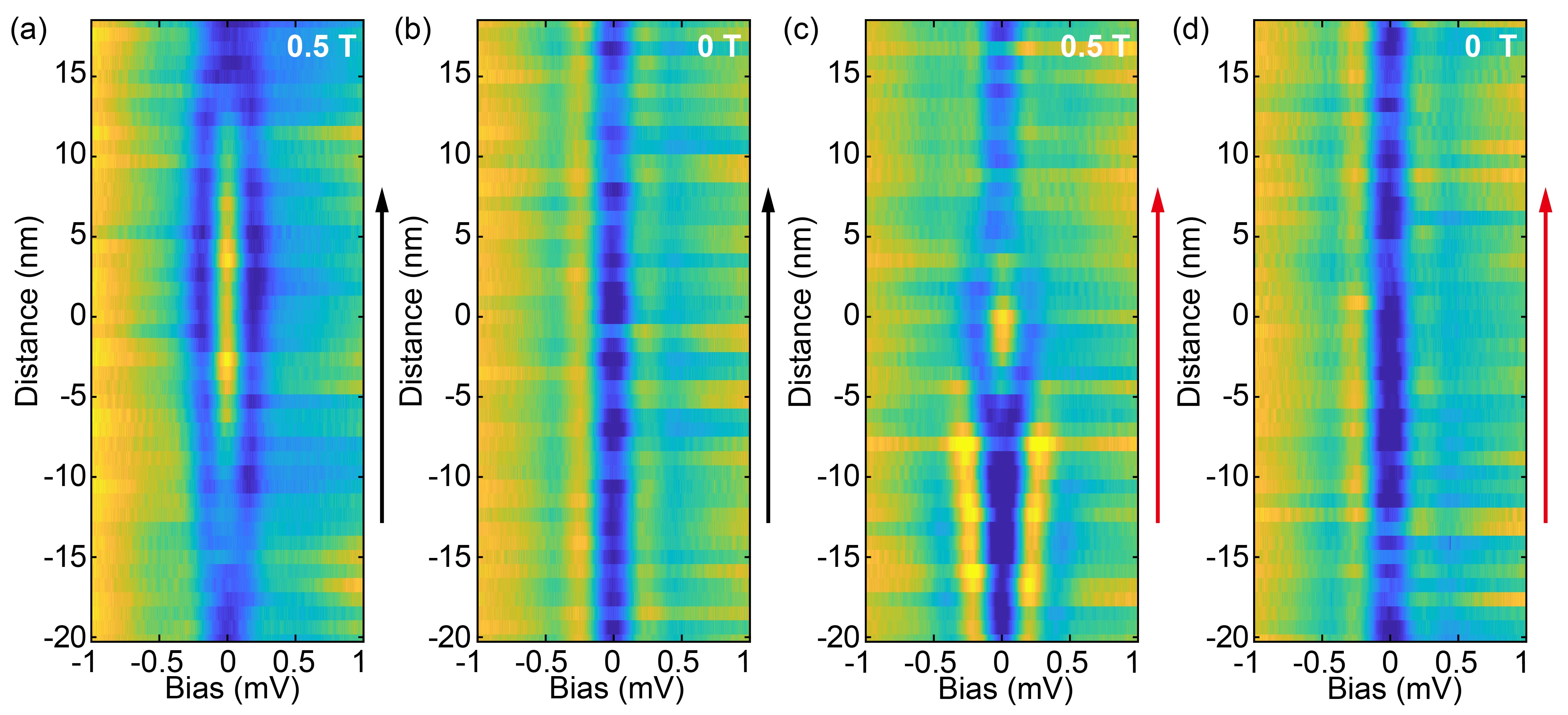


FIG. S5. (a),(c) Linecuts of d*I*/d*V* spectra shown in Figs. 2(e) and 2(f) with 0.5 T magnetic fields. (b),(d) Linecuts of d*I*/d*V* spectra taken with zero magnetic field and along the same lines in (a) and (c), respectively.

1. **Linecuts of d*I*/d*V* spectra taken with different energy ranges**

The local symmetry breaking feature only appears in the energy range of the superconducting gap [Fig. S6(d)]. No such symmetry breaking feature is observed in the d*I*/d*V* spectra taken with larger energy range [Fig. S6(b)]. As shown in the linecut of d*I*/d*V* spectra [Fig. S6(b)], the ~−4.5 mV energy peak has no change.


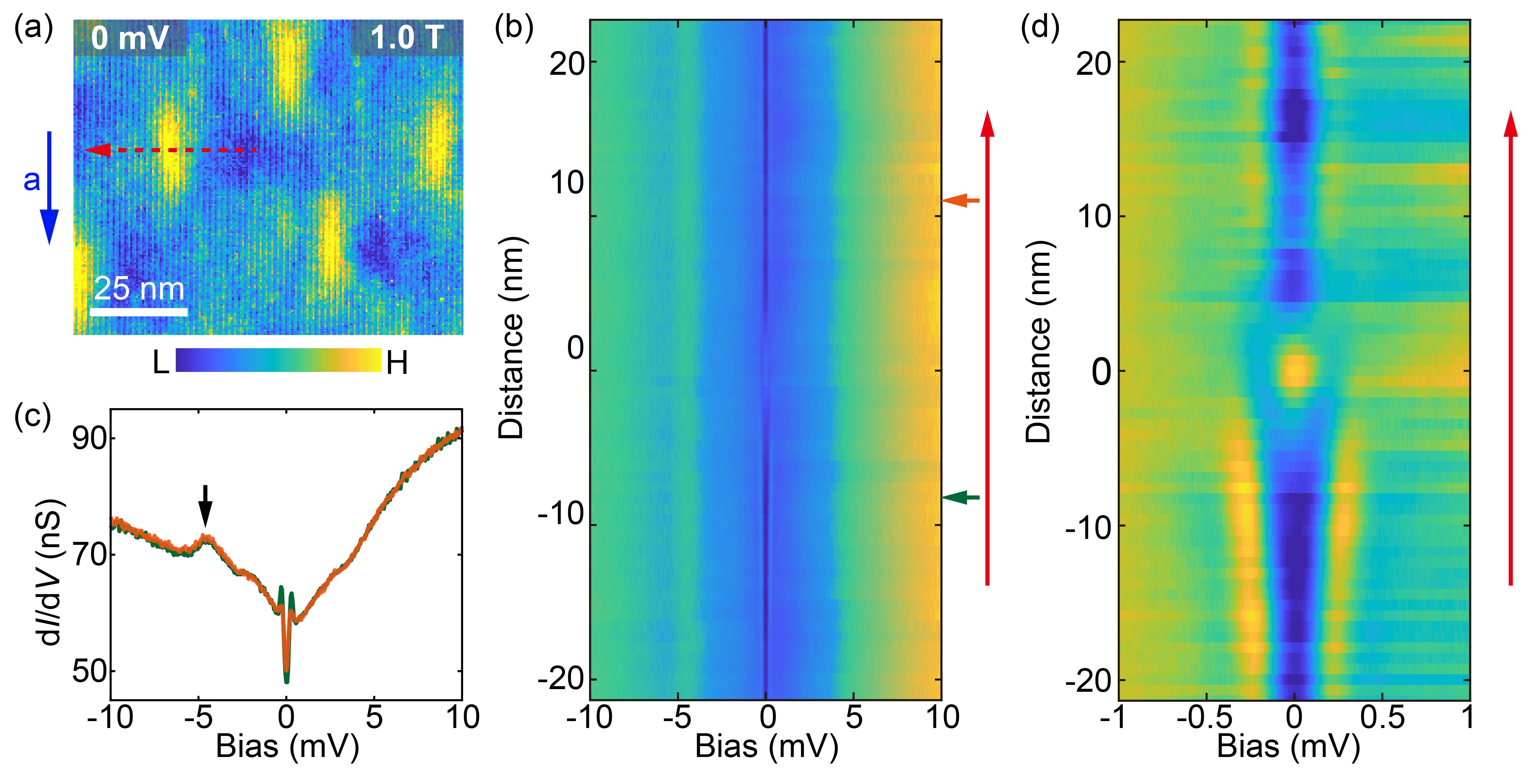


FIG. S6. (a) Zero-energy d*I*/d*V* map taken in a magnetic field *B* = 1 T. (b) d*I*/d*V* linecut profile taken along red dashed arrows in (a). (c) d*I*/d*V* spectra taken on the two sides of the vortex core. The positions are marked by the colored arrows in (b). (d) The same as (b), but taken with ±1 mV energy range.

**References**

1. Jiao L, Howard S, Ran S *et al.* Chiral superconductivity in heavy-fermion metal UTe_2_. *Nature*. 2020; **579**: 523-527.

2. Gu Q, Carroll JP, Wang S *et al.* Detection of a pair density wave state in UTe_2_. *Nature*. 2023; **618**: 921-927.

3. Aishwarya A, May-Mann J, Raghavan A *et al.* Magnetic-field-sensitive charge density waves in the superconductor UTe_2_. *Nature*. 2023; **618**: 928-933.

4. Aishwarya A, May-Mann J, Almoalem A *et al.* Melting of the charge density wave by generation of pairs of topological defects in UTe_2_. *Nature Physics*. 2024; **20**: 964-969.

5. LaFleur A, Li H, Frank CE *et al.* Inhomogeneous high temperature melting and decoupling of charge density waves in spin-triplet superconductor UTe_2_. *Nature Communications*. 2024; **15**: 4456.
